# A Mid-Ventricular Variant of Takotsubo Cardiomyopathy: A Case Study and Review of Literature

**DOI:** 10.7759/cureus.9403

**Published:** 2020-07-26

**Authors:** Gian Lima, Maria Camila Trejo-Paredes, Eduardo Cardoso

**Affiliations:** 1 Internal Medicine, University of Connecticut Health Center, Farmington, USA

**Keywords:** takotsubo, acute heart failure, st-elevation myocardial infarction (stemi), reversible cardiomyopathy, stress-related cardiomyopathy

## Abstract

Different variants of Takotsubo cardiomyopathy (TC) have been described recently. In the present case, we report a post-menopausal woman who had been experiencing significant emotional distress, admitted with typical chest pain, electrocardiographic changes, and elevated troponin levels. She underwent left heart catheterization that demonstrated mild non-obstructive coronary disease and mid-ventricular focal wall motion abnormality, consistent with the mid-ventricular variant of TC. One month after her discharge, a repeated echocardiogram showed preserved ejection fraction and no wall motion abnormalities. In the mid-ventricular variant, we usually observe a unique end-systolic appearance that resembles a Greek vase. It is possible that atypical patterns of left ventricle (LV) dysfunction related to TC are being underrecognized. Therefore, this case study highlights the importance of recognizing less frequent variants of TC.

## Introduction

Defined as a transient and acute regional wall motion abnormality with non-obstructive coronary artery disease on angiography, Takotsubo cardiomyopathy (TC) was first described by Sato et al. in 1990 [1]. The syndrome was called "Takotsubo” (a Japanese term which means “octopus trap”) after the authors compared the appearance of the left ventricle at end-systole to the local Japanese fishermen’s octopus pots in the Hiroshima fishing markets.

The classical pattern of the left ventricle (LV) regional wall motion abnormality is apical and circumferential mid-ventricular hypokinesia and basal hypercontractility, giving the appearance of virtual "apical ballooning" at end-systole. However, other TC variants have been described recently. In the present case, we describe a post-menopausal woman who had been in significant emotional distress recently, admitted with typical chest pain, who was found to have a rare TC variant on coronary angiography.

## Case presentation

A 60-year-old female with a past medical history significant for hyperlipidemia, tobacco abuse (40 packs/year), bipolar disease, and stage III chronic kidney disease presented to the emergency department (ED) for chest pain evaluation.

The patient stated that she woke up with severe chest pain, which she described as an intense pressure-like sensation radiating to her jaw. The patient also reported nausea and shortness of breath associated with the pain, and there were no aggravating or relieving factors. Approximately one hour after the onset of her pain, the emergency medical service team arrived, and she was given 0.4 mg of nitroglycerin sublingual with the resolution of her symptoms. In the emergency department, she denied persistent chest pain. The patient also mentioned that she had been experiencing significant emotional stress due to financial problems as she had recently been fired from her job, aggravated by her husband undergoing treatment for non-Hodgkin's lymphoma.

**Figure 1:**
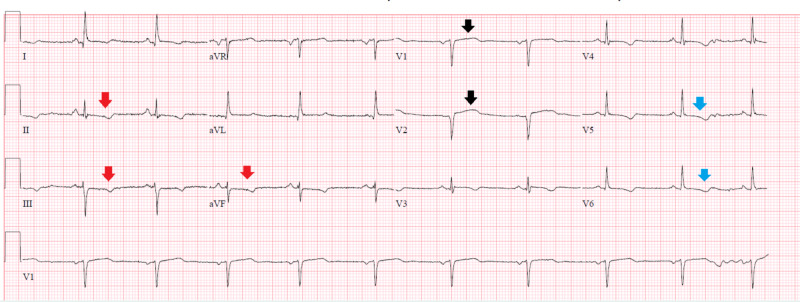
Electrocardiogram on admission Electrocardiogram showed a subtle ST-segment elevation of less than one millimeter in leads V1 and V2 (black arrows), with ST-T changes in leads V5, V6 (blue arrows), and inferior leads II, III, and aVF (red arrows).

In the ED, the patient's initial vital signs were within normal limits, and the physical exam was non-revealing. The initial workup was significant for a mild increase of troponin level at 0.41 ng/mL (reference <0.30 ng/mL), white cell count of 14.1 thous/uL, and creatinine of 1.3 mg/dL, which was her baseline renal function. Electrocardiogram (ECG) showed a subtle ST elevation in V1 and V2, and ST-T changes in the lateral and inferior wall (Figure 1).

**Figure 2:**
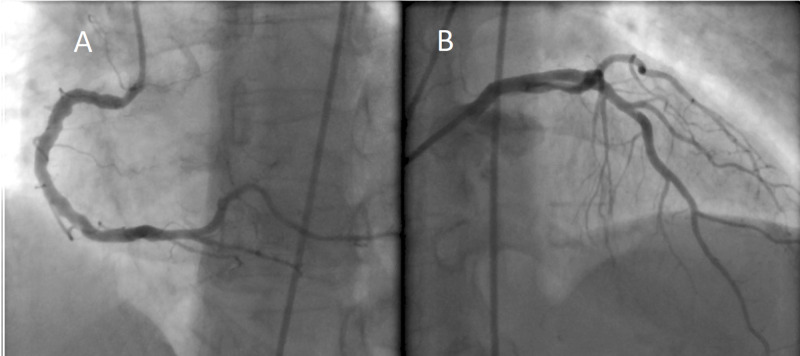
Coronary angiography Left heart catheterization demonstrated no evidence of significant stenosis in the right (A) and left (B) coronary arteries.

**Figure 3:**
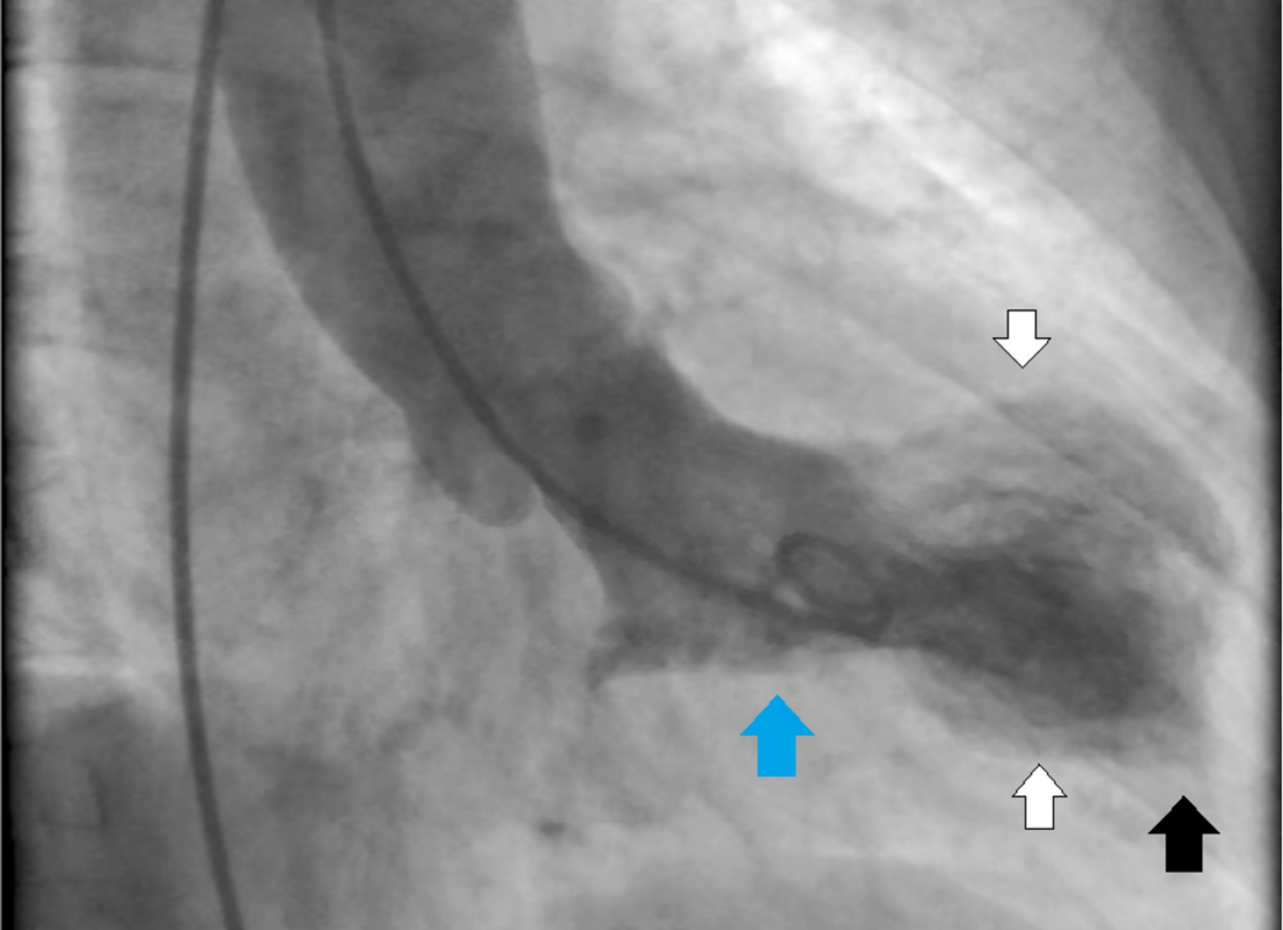
Left ventriculography Mid-ventricular variant of Takotsubo cardiomyopathy: ventriculography showed normal basal (blue arrow) and apical (black arrow) wall motion and hypokinesis of mid-cavitary (white arrows) walls.

The patient was subsequently admitted and placed on anticoagulation with heparin drip and dual anti-platelet therapy for the presumed acute coronary syndrome. A transthoracic echocardiogram showed an ejection fraction of 45%, with hypokinesis of the mid-apical anteroseptal and anterolateral myocardium. The next day, the patient underwent a left heart catheterization that demonstrated mild non-obstructive coronary disease (Figure 2), and ventriculography (Figure 3) showed preserved basal and apical wall motion but significant hypokinesis of mid-cavitary walls, consistent with mid-ventricular variant Takotsubo cardiomyopathy (TC).

The patient remained asymptomatic, with no recurrence of chest pain or dyspnea since admission. She was discharged on aspirin and statins, given the presence of mild non-obstructive coronary artery disease and her risk factors. One month after her discharge, a repeated echocardiogram showed preserved ejection fraction and no wall motions abnormalities.

## Discussion

In the present case, we describe a post-menopausal woman who had been experiencing significant emotional distress, admitted with typical chest pain, ECG changes, and elevated troponin levels. She underwent left heart catheterization that demonstrated mild non-obstructive coronary disease and mid-ventricular focal wall motion abnormality, consistent with mid-ventricular variant TC. 

Epidemiology

It is estimated that TC is responsible for approximately 1.2% of the patients presenting with acute coronary syndrome symptoms and elevated troponin levels with age and female gender being the most important risk factors [2]. In the International Takotsubo Registry (a consortium of 26 centers in Europe and the United States) of 1,750 patients with stress cardiomyopathy, 89.9% were women, and mean age was 66.4 years [3]. 

The syndrome has also been called "broken heart syndrome", as it was often noted to be associated with significant emotional distress (such as bad news involving the death of a loved one, car accidents, natural disasters, strenuous physical effort). However, we believe that this nomenclature is not appropriate as not all patients with TC report a specific trigger. In fact, in the International Takotsubo Registry, 28.5% of patients had no trigger, while 27.7% reported an emotional event, 36% had a physical trigger, and 7.8% reported both physical and emotional triggers. Another reason to avoid the "broken heart" term is the recent recognition that positive emotional distress after pleasant events can also lead to TC, a condition that was named as "happy heart syndrome" [4]. 

Clinical features and diagnosis

The most common clinical presentation in TC is typical chest pain and dyspnea mimicking acute coronary syndrome. However, it is important to highlight other possible symptoms recently described, such as syncope, asthenia, arrhythmias, including ventricular tachycardia and ventricular fibrillation, and even sudden death [5-8]. TC can also be classified as "primary" when it occurs in individuals admitted due to the symptoms related to TC itself, or "secondary" when patients initially admitted for other reasons develop the syndrome in the hospital, often after a surgery or in the setting of severe illness.

The proposed Mayo Clinic criteria require all the following items: 1. transient left ventricular systolic dysfunction (hypokinesis, akinesis, or dyskinesia); 2. absence of obstructive coronary disease or angiographic evidence of acute plaque rupture; 3. new electrocardiographic abnormalities (either ST-segment elevation and/or T wave inversion) or modest elevation in troponin level; 4. absence of pheochromocytoma or myocarditis [9].

Pathophysiology

The pathophysiology of TC is complex and still not completely understood. It has been described that in the acute phase the concentration of plasma catecholamines and stress-related neuropeptides are several times higher than those in patients with ST-elevation myocardial infarction, and these levels can remain elevated up to one week after the onset of the symptoms [10]. Moreover, an increased catecholamine level has been demonstrated not only in the circulation but also at the myocardial level, which somehow resembles the pathophysiology of the "neurogenic stunned myocardium", where patients develop transient LV dysfunction following aneurysm-related subarachnoid hemorrhage due to local release of catecholamine from cardiac nerve endings [11-12]. It is also important to note that there is a disparity in the regional distribution of the adrenergic and cholinergic nerves in the heart [13]. This heterogeneous distribution of the sympathetic and parasympathetic receptors might be the cause of the regional wall motion abnormalities seen in TC.

Besides the increased levels of catecholamines, other factors such as endothelial dysfunction, estrogen deficiency, and microvascular spasm also play an important role in the mechanism of this syndrome. Recent studies have shown that endothelial dysfunction is frequent in patients with TC, and it is responsible for an imbalance between vasoconstriction and vasodilation factors [14]. In post-menopause women, reduced estrogen levels induce both endothelial dysfunction and increased sympathetic drive, possibly the reason why this represents the most vulnerable group for TC [15]. To support this theory, Ueyema et al. performed an interesting experimental study in animals and found that the typical LV dysfunction could be prevented by pre-treatment with α- and β-adrenoceptor blockers and estrogen supplementation [16].

Distribution of wall motion abnormalities

The initial definition of Takotsubo cardiomyopathy described the classical pattern of LV regional wall motion abnormalities with apical and circumferential mid-ventricular hypokinesia and basal hypercontractility, giving the appearance of virtual "apical ballooning" at end-systole. This is today considered the typical TC, present in approximately 80% of the cases [3]. More recently, however, different TC variants have been described. The mid-ventricular variant, seen in our patient, has circumferential mid-ventricular hypokinesia with basal and apical hypercontractility. The International Takotsubo Registry found the mid-ventricular variant in only 14.6% of the cases [3]. In these patients, the heart has a unique end-systolic appearance that resembles a Greek vase or the "ace of spades". Other rare variants have been described, including the inverted Takotsubo or basal variant, biventricular apical dysfunction, dysfunction sparing the apical tip, and the isolated RV Takotsubo syndrome [17]. Interestingly, the study that recently demonstrated that pleasant experiences can also cause TC ("happy heart syndrome"), observed a higher frequency of the mid-ventricular variant in this group of patients, representing 35% of the cases [4].

Treatment and prognosis

The treatment for patients with TC should focus mainly on the management of heart failure (with beta-blockers, angiotensin-converting enzyme inhibitors/angiotensin receptor blockers, aldosterone antagonists, and diuresis if volume overload), and other possible complications such as complete heart block. Although most patients will recover LV function within one to four weeks, the risk of severe complications is similar to that in patients with the acute coronary syndrome. In the International Takotsubo Registry, the in-hospital mortality was 4.1% [3].

## Conclusions

It is possible that atypical patterns of LV dysfunction related to TC are being underrecognized. Furthermore, diagnosing TC in a patient with an unusual presentation following a wide range of possible emotional triggers (including negative or positive feelings) can be challenging. This case study highlights the importance of recognizing less frequent variants of TC.
